# Beyond the Mouth: Oral Health-Related Quality of Life and Emotional Loneliness in Later Life and Their Associations With Sleep Quality and Dream Recall

**DOI:** 10.7759/cureus.113322

**Published:** 2026-07-24

**Authors:** Christos Tsironis, Elena Dragioti, Stefanos Mantzoukas, Agni Nakou, Eleni Bartzou, Nektaria Zagorianakou, Fotios Tatsis, Athina Paschou, Mary Gouva

**Affiliations:** 1 Scientific Laboratory of Psychology and Person-Centered Care, University of Ioannina, Ioannina, GRC; 2 Research Laboratory Psychology of Patients, Families & Health Professionals, University of Ioannina, Ioannina, GRC

**Keywords:** biopsychosocial health, dream recall, emotional loneliness, healthy aging, older adults, oral health-related quality of life, person-centered care, sleep quality

## Abstract

Background

Oral health-related quality of life (OHRQoL) is important in healthy aging, but its associations with emotional loneliness, sleep, and dream recall remain unclear.

Objective

To examine these associations in community-dwelling older adults.

Methods

This cross-sectional study included 609 adults aged ≥60 years in Greece. OHRQoL was assessed using the Geriatric Oral Health Assessment Index and emotional loneliness using the three-item Emotional Loneliness subscale of the De Jong Gierveld Loneliness Scale. Sleep quality, sleep sufficiency, daytime napping, and dream recall were assessed using brief self-report items. Pearson correlations and hierarchical regressions adjusted for age and gender were conducted.

Results

Better OHRQoL was associated with lower emotional loneliness (r=−0.329, p<0.001) and with a more favorable subjective sleep appraisal, as reflected by the nearly indistinguishable sleep sufficiency (r=0.146, p<0.001) and sleep quality items (r=0.135, p<0.001; inter-item r=0.989). Better OHRQoL was also associated with less frequent daytime napping (r=−0.105, p<0.01) and more frequent dream recall (r=0.104, p<0.05). Emotional loneliness was associated with lower sleep sufficiency and poorer sleep quality, but not with dream recall. In adjusted models, OHRQoL was associated with better sleep quality (β=0.101, p=0.018) and showed a small nominal association with dream recall (β=0.091, unadjusted p=0.032). Emotional loneliness was associated with poorer sleep quality (β=−0.182, p<0.001). Gender was associated with both outcomes, with women reporting poorer sleep quality but more frequent dream recall. No correction for multiple comparisons was applied; therefore, the dream recall finding should be regarded as exploratory and susceptible to Type I error.

Conclusion

The clearest finding was the association between better OHRQoL and lower emotional loneliness. The association with sleep quality was smaller, whereas the dream recall finding was very weak, based on a single nonvalidated item, and may reflect measurement error or Type I error. These cross-sectional findings support a cautious biopsychosocial view of OHRQoL but do not permit causal or clinical conclusions. Replication using validated measures, prespecified analyses, and multiple-testing correction is required.

## Introduction

Population aging has shifted attention from the absence of disease toward the preservation of functional ability, psychological well-being, social participation, and quality of life in later life [1]. Within this broader perspective, oral health is increasingly understood as more than the presence or absence of dental disease. Contemporary frameworks conceptualize oral health as a multidimensional domain encompassing physical, psychological, and social functioning [2,3]. Oral health-related quality of life (OHRQoL) captures the perceived effects of oral conditions on everyday functioning, comfort, emotional well-being, and social participation, thereby complementing traditional clinical indicators [4,5].

Oral difficulties may affect chewing, speaking, smiling, facial expression, and confidence in interpersonal situations. Previous research has associated poorer oral health or OHRQoL with functional decline, depressive symptoms, psychological distress, shame, reduced resilience, lower self-care engagement, and social withdrawal among older adults [6-11]. Taken together, this evidence establishes the psychosocial relevance of OHRQoL without implying that oral health alone determines these broader outcomes.

Sleep represents another multidimensional component of healthy aging and is shaped by interacting biological, psychological, and social factors [12,13]. Sleep disturbances are common in later life and have been associated with chronic health conditions, emotional distress, cognitive difficulties, and reduced quality of life [14,15]. Daytime napping is also frequent among older adults and may reflect age-related, behavioral, compensatory, or broader health-related processes [16]. Its interpretation therefore requires caution, particularly when information on nap duration, timing, intentionality, and underlying reasons for napping is unavailable. Emotional loneliness, defined as the perceived absence of close emotional attachment and relational security, has consistently been associated with poorer subjective sleep outcomes [17-20]. Beyond its association with sleep, loneliness has also been linked to cognitive decline and an increased risk of dementia in later life, underscoring its importance as a broader adverse health concern among older adults [19]. These findings provide a rationale for examining emotional loneliness alongside OHRQoL and sleep indicators.

Dream recall frequency represents a narrower and less well-established aspect of sleep-related experience. Previous research has examined dream activity in relation to emotional and cognitive processes [21,22], while the frequency of dream recall has been shown to vary according to individual, attentional, and contextual factors [23,24]. It was included in the present study as an exploratory subjective nocturnal variable rather than as an outcome for which a direct oral-health mechanism was hypothesized. We examined whether the associations of OHRQoL with broader sleep-related and psychosocial experiences extended to this additional domain. No direct causal pathway from OHRQoL to dream recall was assumed. Moreover, recall frequency does not capture dream content, emotional tone, vividness, or the broader experience of dreaming.

Although OHRQoL has been linked to psychological and social well-being, and substantial research has examined sleep in older adults, emotional loneliness, subjective sleep indicators, and dream recall frequency have rarely been studied together in relation to oral health. Guided by the biopsychosocial model [25] and the literature reviewed above, the following theory-guided hypotheses were examined: (H1) better OHRQoL would be associated with lower emotional loneliness; (H2) better OHRQoL would be associated with greater sleep sufficiency, better subjective sleep quality, and less frequent daytime napping; (H3) greater emotional loneliness would be associated with poorer subjective sleep quality and lower sleep sufficiency; and (H4) OHRQoL would remain positively associated with sleep quality after adjustment for age, gender, and emotional loneliness. Because evidence concerning OHRQoL and dream recall was limited and no direct explanatory mechanism was specified, analyses involving dream recall frequency were treated as exploratory rather than hypothesis-testing. These hypotheses were theory-guided but were not preregistered and should therefore not be interpreted as strictly confirmatory.

## Materials and methods

Study design and participants

This study employed a cross-sectional design to investigate the associations between OHRQoL, emotional loneliness, sleep experiences, and dream recall among older adults.

Participants were 609 community-dwelling older adults in Greece, aged 60 years and older. They were recruited through community centers, local health units, and social associations using a non-probability convenience sampling strategy. Approximately 700 community-dwelling older adults were approached for participation, and 609 eligible participants with complete data for the variables examined in the present study were included in the analyses.

Before recruitment, the minimum sample size was estimated using a standard single-population-proportion calculation, assuming a 95% confidence level (two-sided α=0.05), a 5% margin of error, and an expected proportion of 0.50 to represent maximum variability. This calculation yielded a target sample of approximately 384 participants. It was a precision-based recruitment estimate rather than an effect-size-based power analysis for the correlational or regression models. The final analytic sample of 609 participants exceeded this target. No additional exclusion criteria were applied beyond the predefined eligibility criteria, and participants with incomplete data for the variables examined in the present study were not included in the analyses. 

Eligibility criteria included the following: (a) age ≥60 years and (b) the ability to understand the study information, provide informed consent, follow the questionnaire instructions, and complete the questionnaire independently. No standardized cognitive screening instrument was administered; therefore, cognitive status was not formally established.

Ethical approval for the study was obtained from the Ethics Committee of the University of Ioannina (approval number: 27934; date: July 24, 2024). All procedures were conducted in accordance with the principles of the Declaration of Helsinki and the General Data Protection Regulation (GDPR). Participants provided electronic informed consent prior to participation, were assured of anonymity and confidentiality, and retained the right to withdraw from the study at any time without consequence. No sensitive personal data was collected.

Measures

Oral Health-Related Quality of Life (OHRQoL)

OHRQoL was assessed using the Geriatric Oral Health Assessment Index (GOHAI) [26]. The GOHAI is a widely used self-report instrument designed to evaluate OHRQoL in older adults across three domains: physical function (e.g., chewing and speaking), psychosocial function, and pain or discomfort. The scale consists of 12 items assessing functional, psychosocial, and pain-related aspects of oral health. Higher scores indicate better perceived OHRQoL. The GOHAI has demonstrated satisfactory reliability and validity across diverse populations. Cronbach’s α for the present sample was 0.732.

Emotional Loneliness

Loneliness was assessed using the six-item version of the De Jong Gierveld Loneliness Scale [27], a widely used instrument in aging research with well-established psychometric properties. The scale measures two distinct dimensions of loneliness: emotional loneliness, reflecting the absence of close emotional attachment and intimate relationships, and social loneliness, reflecting deficiencies in the broader social network. Subscale scores were calculated according to the recommended scoring procedures, with higher scores indicating greater levels of loneliness. In the present study, emotional loneliness was operationalized using the three-item Emotional Loneliness subscale, with higher scores indicating stronger feelings of emotional isolation and a lack of meaningful emotional connectedness. The three-item Emotional Loneliness subscale demonstrated satisfactory internal consistency in the present sample (Cronbach’s α=0.80). This coefficient was calculated specifically for the three emotional loneliness items included in the analyses, rather than for the full six-item scale.

Sleep Quality and Dream Variables

Subjective sleep and dream-related indicators were assessed using five brief study-specific self-report items. The exact wording was: “Do you think you get enough sleep?” for sleep sufficiency; “Do you think your sleep is good?” for subjective sleep quality; “Do you tend to take naps during the day?” for daytime napping; “Do you remember your dreams?” for dream recall frequency; and “Do you like remembering your dreams?” for attitudes toward dream recall.

The items assessing sleep sufficiency, subjective sleep quality, daytime napping, and dream recall frequency were rated on five-point ordinal scales, with higher scores indicating greater sleep sufficiency, better perceived sleep quality, more frequent daytime napping, and more frequent dream recall, respectively. Attitudes toward remembering dreams were assessed on a three-category scale (no, indifferent, yes).

These items were developed for the purposes of the present study to provide a brief and feasible assessment of subjective sleep and dream experiences in community-based older adults. As they were not part of a previously validated standalone questionnaire, they should be interpreted as brief subjective indicators rather than comprehensive or psychometrically validated measures. Consequently, they may be more susceptible to measurement error and limited construct validity compared to standardized multi-item instruments.

Despite these limitations, similar single-item or brief measures have been widely used in population-based aging research as pragmatic indicators of subjective sleep experience [12,13].

Sociodemographic Characteristics

Information regarding age, gender, educational level, marital status, living arrangements, and chronic disease status was also collected.

Statistical analysis

Data were analyzed using IBM SPSS Statistics for Windows, Version 25 (Released 2017; IBM Corp., Armonk, New York, United States).

Descriptive statistics, including means, standard deviations, frequencies, and ranges, were calculated to characterize the sample and the distributions of the study variables. Pearson product-moment correlation coefficients were computed to examine bivariate associations among oral health-related quality of life, emotional loneliness, sleep sufficiency, subjective sleep quality, daytime napping, dream recall frequency, and age.

Group differences in the primary study variables were examined using independent-samples t-tests and one-way analyses of variance, as appropriate. Effect sizes were considered alongside statistical significance.

Hierarchical multiple regression analyses were conducted to examine whether OHRQoL remained independently associated with subjective sleep quality and dream recall frequency. The two outcomes were examined in separate models. At Step 1, age and gender were entered as demographic covariates. At Step 2, OHRQoL and emotional loneliness were entered simultaneously to evaluate their incremental contribution beyond age and gender. Changes in explained variance (ΔR²), standardized regression coefficients (β), 95% confidence intervals, and corresponding p-values were examined.

Sleep sufficiency was not examined as an additional regression outcome because it was almost perfectly correlated with subjective sleep quality (r=0.989). Separate regression analyses of these two indicators would therefore have been substantively redundant. Subjective sleep quality was retained as the broader global appraisal of sleep, while the bivariate findings for sleep sufficiency were reported descriptively and interpreted cautiously.

Regression analyses were conducted using complete-case data (n=607), following listwise exclusion of participants with missing values in the variables included in the models. Analyses concerning emotional loneliness and subjective sleep quality were conducted in relation to the theory-guided hypotheses stated in the Introduction, whereas analyses involving dream recall frequency were exploratory. Because the hypotheses and analytical distinctions were not preregistered, the findings should not be regarded as strictly confirmatory.

No formal adjustment for multiple comparisons was applied. Accordingly, the reported p-values are unadjusted, and the possibility of Type I error cannot be excluded. Weaker findings, particularly those involving dream recall frequency, were therefore interpreted as exploratory and not as statistically robust evidence of an association. All statistical tests were two-sided, and statistical significance was set at p<0.05.

## Results

Descriptive statistics

The study included 609 community-dwelling older adults. Participants reported a mean OHRQoL (GOHAI) score of 37.27 (SD=7.76). Participants reported mean scores of 3.81 for both sleep sufficiency (SD=1.09) and subjective sleep quality (SD=1.08). Daytime napping was relatively common (M=3.63, SD=1.20), while dream recall was reported at a moderate frequency (M=3.00, SD=1.12). Detailed descriptive statistics are presented in Table 1.

**Table 1:** Descriptive statistics of oral health, loneliness, sleep, and dream variables (N=609)

Variable	Mean	SD	Minimum	Maximum
Oral Health-Related Quality of Life (GOHAI)	37.27	7.76	12	60
Emotional Loneliness	2.81	2.19	0	6
Sleep Sufficiency	3.81	1.09	1	5
Sleep Quality	3.81	1.08	1	5
Daytime Napping	3.63	1.2	1	5
Dream Recall	3	1.12	1	5

Associations between oral health, loneliness, sleep, and dream recall

Bivariate correlation analyses revealed a consistent pattern linking OHRQoL with both psychosocial and nocturnal indicators (Table 2).

**Table 2:** Correlations between oral health-related quality of life, loneliness, sleep, and dream variables GOHAI: Geriatric Oral Health Assessment Index; ^*^p<0.05, ^**^p<0.01, ^***^p<0.001. Reported p-values are unadjusted. No formal correction for multiple comparisons was applied; weaker associations should therefore be interpreted cautiously. Sleep sufficiency and subjective sleep quality were nearly perfectly correlated and should not be interpreted as empirically distinct indicators in this sample.

Variable	1	2	3	4	5	6
1. GOHAI	—					
2. Emotional Loneliness	-0.329^***^	—				
3. Sleep Sufficiency	0.146^***^	-0.221^***^	—			
4. Sleep Quality	0.135^***^	-0.215^***^	0.989^***^	—		
5. Daytime Napping	-0.105^**^	-0.021	0.321^***^	0.313^***^	—	
6. Dream Recall	0.104^*^	-0.036	0.178^***^	0.178^***^	0.123^**^	—
7. Age	-0.229^***^	0.303^***^	0.02	0.019	0.184^***^	-0.027

Better OHRQoL was associated with lower emotional loneliness (r=-0.329, p<0.001), representing the most substantial association among the primary study variables. In addition, higher GOHAI scores were associated with greater sleep sufficiency (r=0.146, p<0.001), better sleep quality (r=0.135, p<0.001), less frequent daytime napping (r=-0.105, p<0.01), and more frequent dream recall (r=0.104, p<0.05).

Emotional loneliness demonstrated a significant negative relationship with both sleep sufficiency (r=-0.221, p<0.001) and sleep quality (r=-0.215, p<0.001), suggesting that older adults experiencing greater emotional disconnection also reported less favorable sleep experiences. Notably, emotional loneliness was not significantly associated with dream recall frequency.

Age was negatively associated with oral health-related quality of life (r=-0.229, p<0.001) and positively associated with emotional loneliness (r=0.303, p<0.001) and daytime napping (r=0.184, p<0.001). No significant relationship emerged between age and dream recall.

Sleep sufficiency and subjective sleep quality were nearly perfectly correlated (r=0.989). Among the 608 participants with complete responses to both items, 597 (98.2%) selected exactly the same response category, while the remaining 11 differed by only one category. The two indicators were therefore not empirically distinguishable in this sample and were interpreted as highly overlapping measures of a single subjective sleep appraisal rather than as separate sleep dimensions. Subjective sleep quality was retained as the sleep outcome in the multivariable analysis because it represented the broader global appraisal of sleep; conducting a parallel regression analysis for sleep sufficiency would have been substantively redundant.

Hierarchical regression analysis predicting sleep quality

A hierarchical multiple regression analysis was conducted to examine whether OHRQoL and emotional loneliness were independently associated with sleep quality after controlling for age and gender.

In the first step, age and gender explained a modest proportion of variance in sleep quality (R²=0.011). Gender emerged as a significant predictor, whereas age was not significantly associated with the outcome.

Following the addition of OHRQoL and emotional loneliness in the second step, the explained variance increased significantly to 6.1% (ΔR²=0.050, p<0.001). Better OHRQoL was independently associated with better subjective sleep quality (β=0.101, p=0.018), whereas greater emotional loneliness was independently associated with poorer subjective sleep quality (β=−0.182, p<0.001). Gender also remained independently associated with sleep quality, with women reporting poorer subjective sleep quality than men, while age was not significantly associated with the outcome. Emotional loneliness showed the strongest standardized association in the final model, whereas the standardized associations of gender and OHRQoL were broadly comparable in magnitude (Table 3).

**Table 3:** Hierarchical regression analysis predicting sleep quality GOHAI: Geriatric Oral Health Assessment Index; B: unstandardized regression coefficient; SE: standard error; β: standardized regression coefficient; 95% CI: 95% confidence interval for B; ΔR²: change in explained variance.

Predictor	B	SE	β	t	95% CI for B	p
Model 1 (Covariates)						
Constant	4.166	0.431	–	9.669	3.320, 5.012	<0.001
Age	0.001	0.005	0.004	0.097	−0.010, 0.011	0.922
Gender	−0.241	0.091	−0.107	−2.645	−0.420, −0.062	0.008
Model 1 statistics: N = 607; R² =0.011; Adjusted R² =0.008 F(2, 604) = 3.511; p=0.031						
Model 2 (Adding GOHAI and Emotional Loneliness)						
Constant	3.082	0.513	–	6.001	2.073, 4.090	<0.001
Age	0.01	0.005	0.082	1.96	−0.000, 0.021	0.05
Gender	−0.215	0.09	−0.095	−2.398	−0.391, −0.039	0.017
GOHAI	0.014	0.006	0.101	2.374	0.002, 0.026	0.018
Emotional Loneliness	−0.090	0.022	−0.182	−4.190	−0.133, −0.048	<0.001
Model 2 statistics: N = 607; R²=0.061 Adjusted R²=0.055; ΔR²=0.050 F(4, 602) =9.756; p<0.001						

These findings suggest that OHRQoL contributes to sleep quality beyond basic demographic characteristics and that emotional loneliness represents an important psychosocial correlate of nocturnal well-being.

Hierarchical regression analysis predicting dream recall

A second hierarchical regression analysis was conducted with dream recall frequency as the dependent variable. At the first step, age and gender accounted for 1.2% of the variance in dream recall frequency (R²=0.012). Gender was significantly associated with dream recall frequency, whereas age was not.

After OHRQoL and emotional loneliness were entered at Step 2, the final model was statistically significant overall (R²=0.021, F(4, 602) = 3.150, p=0.014). However, the model explained only 2.1% of the total variance in dream recall frequency, and the variables added at Step 2 jointly accounted for an additional 0.9% (ΔR²=0.009). This incremental contribution was very small and of uncertain practical relevance. OHRQoL showed a small positive association with dream recall frequency (β=0.091, unadjusted p=0.032), whereas emotional loneliness was not significantly associated with the outcome (β=−0.004, p=0.934) (Table 4).

**Table 4:** Hierarchical regression analysis predicting dream recall B: unstandardized regression coefficient; SE: standard error; β: standardized regression coefficient; 95% CI: 95% confidence interval for B; ΔR²: change in explained variance. Reported p-values are unadjusted. No formal correction for multiple comparisons was applied. The association between oral health-related quality of life and dream recall frequency should therefore be interpreted as exploratory. The association between oral health-related quality of life and dream recall frequency met only the conventional nominal significance threshold and should be regarded as exploratory.

Predictor	B	SE	β	t	95% CI for B	p
Model 1 (Covariates)						
Constant	3.058	0.444	–	6.888	2.186, 3.930	<0.001
Age	−0.006	0.005	−0.043	−1.068	−0.016, 0.005	0.286
Gender	0.232	0.094	0.101	2.477	0.048, 0.417	0.014
Model 1 statistics: N=607; R²=0.012; Adjusted R²=0.009 F(2, 604)=3.704; p=0.025						
Model 2 (Adding GOHAI and Emotional Loneliness)						
Constant	2.355	0.541	–	4.356	1.293, 3.417	<0.001
Age	−0.003	0.006	−0.021	−0.499	−0.014, 0.008	0.618
Gender	0.223	0.094	0.096	2.363	0.038, 0.408	0.018
GOHAI	0.014	0.006	0.091	2.146	0.001, 0.026	0.032
Emotional Loneliness	−0.002	0.023	−0.004	−0.083	−0.047, 0.043	0.934
Model 2 statistics: N=607; R²=0.021; Adjusted R²=0.014; ΔR²=0.009 F(4, 602)=3.150; p=0.014						

Because no adjustment for multiple comparisons was applied, the OHRQoL finding met only the conventional nominal significance threshold and should be regarded as fragile and exploratory rather than as statistically robust evidence of an independent association.

Gender remained independently associated with dream recall frequency, with women reporting more frequent dream recall than men. Its standardized association was slightly larger than that of OHRQoL, although both associations were small. Thus, OHRQoL was neither the predominant nor a practically substantial correlate in this model.

The pattern of findings differed from that observed for sleep quality. Whereas emotional loneliness was closely linked to subjective sleep experiences, dream recall appeared to be associated specifically with OHRQoL rather than with feelings of emotional loneliness. The pattern of findings differed from that observed for sleep quality. Dream recall frequency showed a weak association with OHRQoL but was not associated with emotional loneliness. Given the small effect size, the limited variance explained, the use of a single nonvalidated item, and the absence of adjustment for multiple testing, this finding should be regarded as exploratory. It does not support conclusions about the broader experience, content, or psychological function of dreaming.

## Discussion

**Figure 1:**
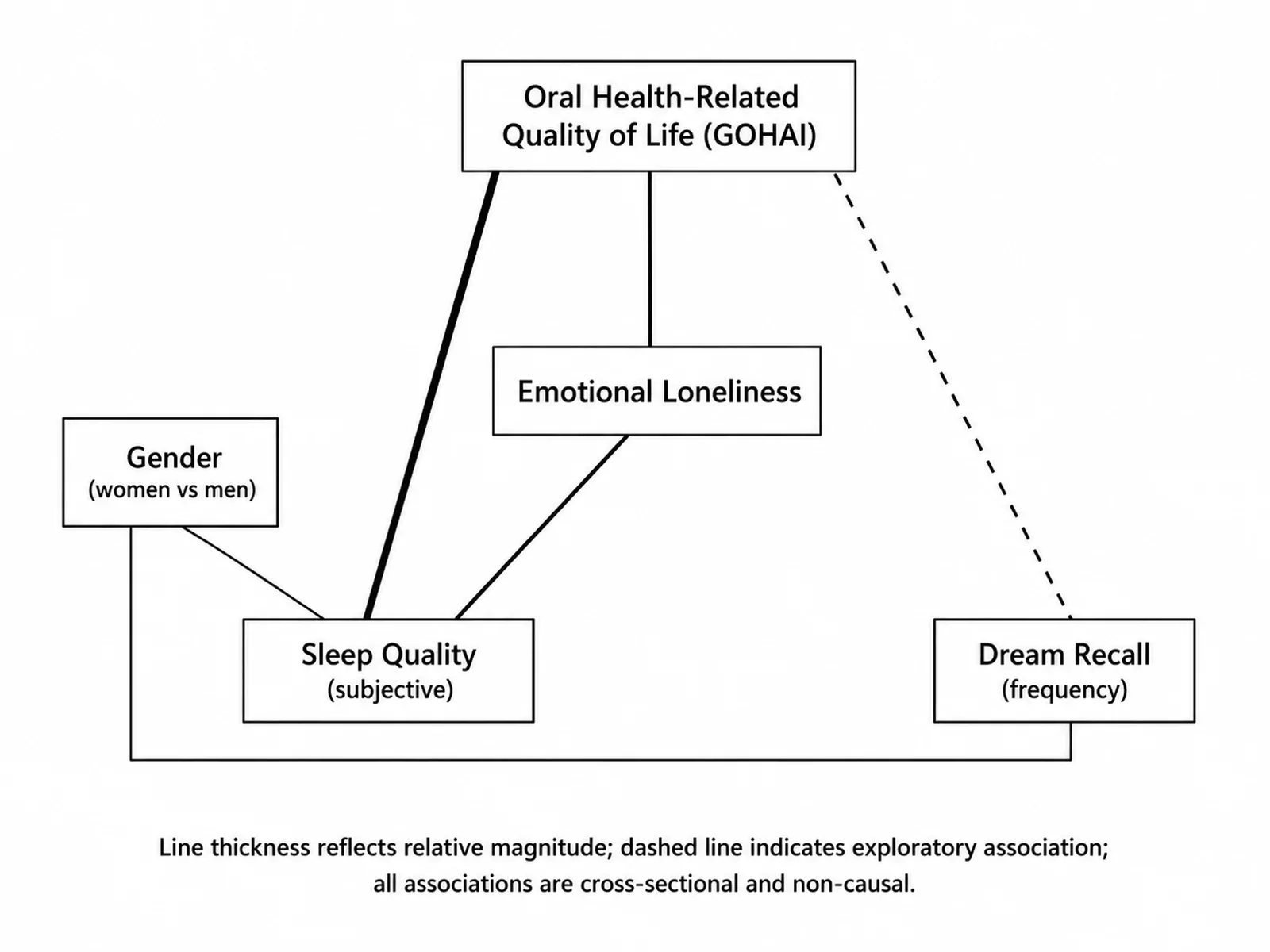
Associative framework summarizing the main observed cross-sectional relationships GOHAI: Geriatric Oral Health Assessment Index; Better oral health-related quality of life was associated most clearly with lower emotional loneliness, while smaller associations were observed with better subjective sleep quality and more frequent dream recall. Emotional loneliness was also associated with poorer subjective sleep quality. Gender was associated with both sleep quality and dream recall frequency, with women reporting poorer sleep quality but more frequent dream recall than men. Line thickness reflects the relative magnitude of the observed associations, and the dashed line denotes the weak, nominal, and exploratory association with dream recall. The lines are nondirectional and do not represent causal, temporal, mediational, or otherwise confirmed pathways. Image credit: Created by the authors using ChatGPT (OpenAI, California, US) and edited in Microsoft Paint (Microsoft Corp., Redmond, US).

The present study examined associations among OHRQoL, emotional loneliness, subjective sleep indicators, and dream recall frequency in community-dwelling older adults. Several principal findings emerged. Better OHRQoL showed its clearest association with lower emotional loneliness. Smaller bivariate associations were observed with better subjective sleep appraisal, less frequent daytime napping, and more frequent dream recall. However, sleep quality and sleep sufficiency were almost perfectly correlated and therefore cannot be regarded as empirically distinct sleep constructs in this sample. Emotional loneliness was associated with poorer subjective sleep but was unrelated to dream recall frequency. Figure 1 provides a descriptive associative summary of the main cross-sectional relationships observed in the present study.

It is not intended to represent a tested directional, causal, or mediational model.

In the adjusted models, OHRQoL remained independently associated with sleep quality and showed a small nominal association with dream recall frequency. Nevertheless, the magnitude of these associations was small, and the additional variance explained was modest, particularly for dream recall. Gender was also independently associated with both outcomes: women reported poorer subjective sleep quality but more frequent dream recall than men. In the sleep quality model, the standardized association of gender was broadly comparable in magnitude to that of OHRQoL, whereas in the dream recall model it was slightly larger. These results indicate that demographic differences were at least as relevant as OHRQoL for some of the examined outcomes.

Among the primary study variables, the strongest observed association was between OHRQoL and emotional loneliness. This finding is consistent with previous research linking oral health with psychosocial functioning and subjective well-being in later life [2,3,9]. Difficulties involving speaking, chewing, smiling, appearance, or comfort may coexist with reduced interpersonal engagement, lower self-confidence, and greater emotional disconnection. Nevertheless, the cross-sectional design does not establish whether poorer OHRQoL precedes emotional loneliness, whether loneliness affects perceptions or management of oral health, or whether both reflect shared psychological, medical, or social factors. The findings therefore support a relational interpretation of OHRQoL but do not demonstrate a causal pathway.

The distinction between emotional and social loneliness is important when interpreting this finding. Emotional loneliness concerns the perceived absence of close emotional attachment and meaningful emotional connectedness, whereas social loneliness refers more broadly to limitations in social networks and social integration [28,29]. The present results specifically concern emotional loneliness and should not be generalized to social loneliness, objective social isolation, or loneliness as a broader construct.

The association between emotional loneliness and poorer subjective sleep is consistent with evidence linking loneliness with disturbed sleep and reduced psychological well-being [17,19]. Emotional loneliness may coexist with psychological distress, emotional arousal, reduced feelings of security, and difficulty experiencing restorative sleep [17-20]. From a biopsychosocial perspective, subjective sleep quality may reflect not only physiological processes but also the emotional and relational context in which individuals live [12,13,25]. In the present study, emotional loneliness remained independently associated with sleep quality after accounting for age, gender, and OHRQoL. However, the effect was modest, and no causal or temporal conclusion can be drawn.

Disturbed sleep in later life is multifactorial and may be associated with psychological distress, medical conditions, pain, medication use, primary sleep disorders, environmental influences, and other factors not assessed in the present study. OHRQoL should therefore be regarded as one possible correlate among many rather than as a general explanation for sleep disturbance. In addition, the near-perfect correlation between sleep quality and sleep sufficiency indicates that participants did not meaningfully differentiate between these two single-item indicators. Their similar associations should therefore not be interpreted as replication across two distinct dimensions of sleep.

Gender was independently associated with both subjective sleep quality and dream recall frequency. Women reported poorer sleep quality but more frequent dream recall than men. These differences may reflect a combination of biological, psychological, social, behavioral, and reporting-related factors. However, the study was not designed to examine gender-related mechanisms, and potentially relevant explanatory variables were not assessed. The gender findings should therefore be regarded as descriptive and hypothesis-generating rather than as evidence supporting a particular explanation.

The association between OHRQoL and dream recall frequency requires substantial caution. Although the coefficient met the conventional unadjusted significance threshold after adjustment for age, gender, and emotional loneliness (β=0.091, p=0.032), the final model explained only 2.1% of the variance in dream recall frequency. The addition of OHRQoL and emotional loneliness at the second step jointly increased the explained variance by only 0.9%; this value should not be interpreted as the unique contribution of OHRQoL alone. These values indicate a very small effect with uncertain practical relevance. Given the limited explanatory power, the single nonvalidated measure of dream recall, the absence of correction for multiple comparisons, and the possibility of residual confounding, the finding is likely to be sensitive to minor changes in sample composition or model specification and may not replicate. It should therefore be considered a fragile, nominal, and hypothesis-generating observation rather than evidence of a reliable theoretical or clinically meaningful relationship.

Dream recall was assessed using a single study-specific item without established psychometric reliability or construct validity. The study did not assess dream content, vividness, emotional tone, distress, symbolism, or the broader subjective experience of dreaming. Given the limited measurement approach and the very small amount of explained variance, the observed association cannot be distinguished confidently from measurement error, individual differences in memory or reporting, residual confounding, or chance. Measurement error alone could plausibly account for the finding. It should therefore not be interpreted as evidence of a reliable, clinically meaningful, or theoretically established relationship. The result is preliminary and hypothesis-generating and requires independent replication using validated dream measures, prespecified analyses, appropriate control for multiple testing, and more comprehensive adjustment for psychological, cognitive, medical, and pharmacological factors.

Taken together, the findings are compatible with a multidimensional understanding of OHRQoL within healthy aging. Previous studies have identified associations between oral health, psychological well-being, self-care behaviors, resilience, and psychopathological symptoms [10,11]. The present findings extend this literature by examining OHRQoL alongside emotional loneliness and subjective sleep-related indicators. They should not, however, be interpreted as demonstrating that oral health determines emotional well-being, sleep quality, or dream recall. Rather, these domains may coexist within a broader biopsychosocial context in which physical, psychological, cognitive, and relational experiences interact [25].

Potential biological pathways may involve oral biofilm, periodontal inflammation, microbial processes, and associated systemic inflammatory burden [2,3]. However, the present study assessed perceived OHRQoL rather than clinical periodontal status, dental biofilm, oral infection, or inflammatory biomarkers. It therefore provides no direct evidence that biological or inflammatory mechanisms contributed to the observed associations.

From a clinical and public health perspective, OHRQoL may be considered alongside emotional and sleep-related concerns when assessing the broader well-being of older adults. The findings do not demonstrate that oral health promotion or dental treatment would reduce loneliness or improve sleep. Longitudinal and intervention studies would be required before such benefits could be inferred. Oral healthcare remains important because of its established functional, nutritional, communicative, and quality-of-life relevance, irrespective of whether it directly alters psychological or sleep-related outcomes.

Overall, the clearest finding was the association between OHRQoL and emotional loneliness. Associations involving subjective sleep were smaller, and sleep quality and sleep sufficiency did not demonstrate empirical distinctiveness. The association with dream recall frequency was weak and exploratory, while gender was at least as relevant as OHRQoL in some of the adjusted models. Given the cross-sectional design, modest effect sizes, reliance on self-report measures, potential residual confounding, and multiple statistical tests, the weaker findings require cautious interpretation and independent replication.

In later life, oral health extends beyond chewing, speaking, and freedom from pain. It is also embedded in communication, self-presentation, social participation, and subjective quality of life. The present findings indicate that oral health-related experiences were associated most clearly with emotional connectedness, whereas their smaller relationships with subjective sleep quality and dream recall remain uncertain and require further study.

Implications for person-centered care and preventive practice

The present findings have implications for person-centered care and preventive health practice, although these implications should be considered in light of the modest effect sizes and the cross-sectional design. Traditionally, oral health interventions have focused primarily on the prevention and treatment of dental disease. OHRQoL, however, also encompasses the perceived functional, psychological, and social consequences of oral conditions [2,3]. Its assessment may therefore contribute to a broader understanding of how older adults experience their health and everyday lives.

From a person-centered perspective, discussion of oral health may provide an opportunity to explore difficulties related to communication, confidence, social participation, emotional well-being, and quality of life. Clinicians should not interpret poor OHRQoL as evidence that an older person is necessarily lonely or experiencing sleep problems. Nevertheless, when oral difficulties are accompanied by social withdrawal, emotional distress, or sleep complaints, broader assessment or appropriate referral may be warranted. Comprehensive assessment can help identify coexisting needs without assuming that one domain has caused another.

The association between OHRQoL and emotional loneliness highlights the importance of considering relational and social experiences alongside physical care. Oral health difficulties may coexist with embarrassment, communication problems, reduced confidence, concern about appearance, and limitations in social participation. Emotional loneliness or reduced social engagement may likewise coexist with fewer resources, less motivation, or less support for maintaining oral health. However, these possible explanations were not directly tested, and the direction or temporal ordering of the observed relationship cannot be determined from the present study. Oral function and social participation should therefore be regarded as potentially interrelated dimensions rather than as components of a demonstrated causal sequence.

The findings do not establish that improving oral health will improve sleep quality or alter dream recall frequency. The observed sleep associations were small, while the dream recall association was particularly weak and based on a single-item measure. Consequently, dream recall should not currently be used as a clinical indicator of oral health, psychological functioning, or treatment response. Its inclusion in the present study is best understood as exploratory and as identifying a question for further research rather than an immediate target for clinical practice.

At a public health level, interdisciplinary collaboration among dental, primary care, mental health, and gerontological services may support more comprehensive care for older adults. Such collaboration is justified by the multifaceted needs of aging populations and by the established importance of oral health for daily functioning and quality of life, rather than by an assumption that oral health interventions will directly resolve loneliness or sleep difficulties. Health-promotion initiatives can appropriately situate oral health within broader strategies supporting functional ability, participation, emotional well-being, and meaningful aging [2,3].

Ultimately, person-centered care requires attention not only to disease and functional status but also to how individuals experience their bodies, relationships, and everyday lives. OHRQoL forms part of this wider experience and may help clinicians recognize concerns that would otherwise remain unspoken. However, its associations with emotional loneliness and sleep-related outcomes should be interpreted as contextual information rather than as evidence of causation or as a basis for specific psychological or sleep interventions [3-5].

Strengths of the study

The present study has several strengths, including a large community-based sample of older adults, the simultaneous examination of oral health, emotional loneliness, sleep experiences, and dream recall within a single framework, and the use of validated measures of OHRQoL and loneliness. 

Study limitations and future directions

Several limitations should be considered when interpreting the findings. First, the cross-sectional design does not permit conclusions regarding causality, temporal ordering, or directionality. Although OHRQoL was statistically associated with emotional loneliness, sleep quality, and dream recall frequency, the findings do not demonstrate that poorer OHRQoL causes loneliness or sleep-related difficulties. Reverse or reciprocal relationships are also possible, and the observed associations may reflect shared underlying factors.

Second, the associations with the sleep-related variables were generally small. The association between OHRQoL and emotional loneliness was the strongest of the primary associations, whereas those with sleep quality, daytime napping, and dream recall frequency were considerably weaker. The regression models also explained only a modest proportion of outcome variance. Statistical significance in a relatively large sample should not be interpreted as evidence of clinical or practical importance. The practical relevance of the sleep-related findings therefore remains uncertain.

Third, the original sample-size determination was based on a precision-based estimate for a population proportion rather than on a prespecified effect size for the correlational and regression analyses reported in this study. Accordingly, although the final analytic sample exceeded the recruitment target, the calculation should not be interpreted as a formal a priori power analysis for the specific statistical models examined.

Fourth, the association with dream recall frequency was particularly small and was based on a single study-specific item with no established psychometric reliability or construct validity. The final regression model explained only 2.1% of the variance in dream recall frequency, while the variables introduced at the second step jointly increased the explained variance by only 0.9%. These values indicate minimal explanatory value. Given the limited measurement approach and the very small amount of explained variance, the observed association cannot be distinguished confidently from measurement error, individual differences in reporting or memory, residual confounding, sampling variation, or chance. Measurement error alone could plausibly account for the finding. The OHRQoL coefficient may also be unstable and could change or become nonsignificant with minor variations in sample composition, covariate selection, measurement, or model specification. Because no formal robustness or specification-sensitivity analyses were conducted, the stability of this finding cannot be established. In addition, the study did not assess dream content, vividness, emotional tone, distress, symbolism, or other dimensions of dreaming. Consequently, the finding should not be interpreted as evidence concerning the broader experience or psychological function of dreaming. Its theoretical and clinical meaning remains uncertain, and it should be treated solely as preliminary and hypothesis-generating.

Fifth, although age and gender were included as covariates and emotional loneliness was considered in the primary regression models, several potentially important confounding variables were not included in the analyses. These include depressive and anxiety symptoms, medication use, pain, diagnosed sleep disorders, multimorbidity, socioeconomic circumstances, and recent dental treatment. Clinical periodontal status, dental biofilm, specific oral and dental conditions, markers of oral infection, and systemic inflammatory biomarkers were also not assessed. Residual confounding may therefore have contributed to some of the observed associations, and the study cannot determine whether biological or inflammatory processes were involved.

Cognitive functioning was not assessed using a standardized screening instrument. Consequently, milder or unrecognized cognitive impairment may have affected participants’ comprehension, recall, and response reliability, particularly for the single-item measure of dream recall frequency. At the same time, the requirement that participants complete the questionnaire independently may have resulted in the underrepresentation of older adults with more substantial cognitive impairment.

Sixth, several bivariate associations and regression models were examined. Because no formal adjustment for multiple comparisons was applied, the possibility of Type I error and false-positive findings cannot be excluded, particularly for the smaller associations. The weaker findings, especially those involving dream recall frequency, should therefore be interpreted as exploratory and require confirmation in independently recruited samples. Moreover, because the theory-guided expectations and analytical distinctions were not preregistered, the findings should not be regarded as strictly confirmatory.

Finally, participants were community-dwelling older adults recruited in Greece through community centers, local health units, social services, and community associations using a non-probability convenience sampling strategy. Individuals who attend such settings may differ systematically from the wider older adult population. In particular, they may be more socially connected, functionally independent, or engaged with community and healthcare services, although those recruited through health units may also have had greater health needs. The sample may therefore have underrepresented homebound, highly isolated, frail, institutionalized, or cognitively impaired older adults. This selection process may have affected the observed distribution of emotional loneliness and restricted variability in social and health-related characteristics, potentially influencing the magnitude of the reported associations. The direction of this bias cannot be determined from the available data. Consequently, the findings should not be generalized to the broader older adult population or to older adults living in different cultural and healthcare contexts.

Future research should employ longitudinal or prospective designs, validated multidimensional measures of sleep and dreaming, more detailed assessments of daytime napping, and clinical oral and periodontal examinations. More comprehensive adjustment for psychological, medical, pharmacological, cognitive, and socioeconomic factors would also be needed. The inclusion of microbiological or inflammatory biomarkers could further support the investigation of possible biological mechanisms underlying the observed associations. Preregistered hypotheses, prespecified analytical plans, and appropriate correction for multiple testing would strengthen the reliability and interpretability of future findings.

Future studies could additionally examine the emotional tone, imagery, recurring themes, and personally attributed meaning of dreams. Such research might explore whether distressing dreams are experienced as subjective signals of psychological strain or of waking-life concerns requiring attention, including, but not limited to, health and self-care needs. This possibility could not be evaluated in the present study, which assessed only dream recall frequency and did not examine dream content, emotional valence, or distress.

## Conclusions

In this sample of community-dwelling older adults, better OHRQoL was most clearly associated with lower emotional loneliness. Smaller associations were observed with subjective sleep quality and dream recall frequency. Given the cross-sectional design, self-reported measures, modest effect sizes, residual confounding, and the exploratory nature of the analyses, no causal or clinical conclusions can be drawn.

The findings support further investigation of OHRQoL within broader relational and sleep-related contexts in later life. In particular, the weak association with dream recall frequency requires independent replication using validated multidimensional measures before any theoretical or practical significance can be attributed to it.
